# Microbial Diversity and Structure Are Drivers of the Biological Barrier Effect against *Listeria monocytogenes* in Soil

**DOI:** 10.1371/journal.pone.0076991

**Published:** 2013-10-08

**Authors:** Anne-Laure Vivant, Dominique Garmyn, Pierre-Alain Maron, Virginie Nowak, Pascal Piveteau

**Affiliations:** 1 Université de Bourgogne, UMR1347 Agroécologie, Dijon, France; 2 INRA, UMR1347 Agroécologie, Dijon, France; 3 Plateforme GenoSol, INRA, UMR1347 Agroécologie, Dijon, France; University of Wisconsin-Milwaukee, United States of America

## Abstract

Understanding the ecology of pathogenic organisms is important in order to monitor their transmission in the environment and the related health hazards. We investigated the relationship between soil microbial diversity and the barrier effect against *Listeria monocytogenes* invasion. By using a dilution-to-extinction approach, we analysed the consequence of eroding microbial diversity on *L. monocytogenes* population dynamics under standardised conditions of abiotic parameters and microbial abundance in soil microcosms. We demonstrated that highly diverse soil microbial communities act as a biological barrier against *L. monocytogenes* invasion and that phylogenetic composition of the community also has to be considered. This suggests that erosion of diversity may have damaging effects regarding circulation of pathogenic microorganisms in the environment.

## Introduction

Understanding the ecology of pathogenic microorganisms is critical in order to control associated health hazards. The presence of food-borne pathogens in the farm environment increases the risk of transfer to animals and plant raw products and eventually to food. Soil and water environments may be reservoirs of human pathogens such as pathogenic *Escherichia coli* [1-3], *Pseudomonas aeruginosa* [3,4], *Clostridium perfringens* [5], *Staphyloccocus aureus* [3,6] and *Listeria monocytogenes* [7,8]. *L. monocytogenes* which is the causative agent of listeriosis, a serious food-borne infection affecting essentially immuno-compromised individuals, the elderly and pregnant women [9] is found in the environment. It has been isolated from water systems [8,10], vegetation [11], soil [7], farms [12-15], food industries [16-18] and faeces of animals [19-21]. So far, ecosystem characteristics that affect the fate of the pathogen in natural environments are poorly understood. Reports on the survival of *L. monocytogenes* in soil suggest that abiotic edaphic factors such as the clay content or pH of soils as well as biotic factors [7,12,22-24] may affect survival. However, biotic and abiotic factors are intertwined in soils and these reports do not clearly state the contribution of soil microbiota to the fate of *L. monocytogenes* in soil.

Current theories of ecology addressing plants and animals may be relevant to the understanding of the fate of microorganisms introduced in natural environments. Moreover, microbial experimental set-ups may be useful to confirm current ecology theories [25]. Biological invasion is one of these theories. Developed in the fifties, it postulates that high species diversity protects ecosystems from invasion by alien species [26]. Further investigations confirmed this hypothesis and showed that, at small spatial scales, high species diversity of plant and animal communities was an effective biological barrier that decreased invader success [27-31]. A combination of improved resource utilisation limiting nutrient availability and increased competition may explain these observations [27,32]. Reports addressing the role of soil microbial diversity and putative mechanisms in the biological barrier against microbial invasion are limited. Pathogenic *Escherichia coli* [33] and *Pseudomonas aeruginosa* [34] were studied as invading microorganisms, but in these studies, microbial diversity was partially characterised through fingerprinting analyses, which precludes the accurate assessment of microbial diversity parameters.

The main objective of this study was to investigate if, as proposed by the theory of biological invasion, soil microbial species diversity could act as a biological barrier preventing invasion by *L. monocytogenes*. In other words, we addressed the consequences of soil diversity erosion on the fate of a human bacterial pathogen in the telluric environment. We constructed microcosms with similar bacterial abundance but with altered soil microbial diversity through a dilution-to-extinction approach and we characterised the resulting bacterial diversity by high throughput pyrosequencing. The fate of inoculated *L. monocytogenes* was then monitored and survival patterns were analyzed with regards to the taxonomic profiles of the constructed microcosms.

## Materials and Methods

### Soil and soil extract

Soil was sampled in a pasture located in Burgundy. This sampling site belongs to a country-wide soil sampling network (RMQS) based on a 16 x 16 km systematic grid covering the whole of France [35]. Collection of soil sample was performed on private land with the consent of the land owner. Only soil was sampled. Endanger species were not present in the pasture and none were sampled. Twenty-five individual core samples of topsoil (0–30 cm) were taken using a sampling design within an area of 20 x 20 m. The core samples were then mixed to obtain a composite sample. The soil sample was then sieved to 5 mm. Aliquots of the soil were treated by γ-ionization (45 KGy minimum) by Ionisos (Dagneux, France). Soil’s attributes such as location, pedology, chemistry and land use are stored in the DONESOL database [36]. Briefly, it is a clay soil with neutral pH. Organic carbon and nitrogen content were respectively 35.3 and 3.9 g.kg^-1^.

γ-sterilised soil extract was prepared according to Pochon and Tardieux method with some modifications. Five hundred g of soil were mixed in 750 ml of water for 30 minutes at 120 rpm and autoclaved 1 hour at 130°C. Soil suspensions were centrifuged at 10 000 g for 20 minutes and supernatants were filtered on Whatman paper (Sigma-Aldrich, Saint-Quentin Fallavier, France). The particle-free soil extract obtained was used as sterile diluent after autoclaving (20 minutes, 120°C).

### Dilution-to-extinction setup and microcosms preparation

One hundred g of soil were blended in 300 ml of water for 1.5 minutes in a waring blender. The soil suspension was serially diluted in sterilised soil extract. The undiluted, 10^2^-diluted and 10^4^-diluted suspensions were inoculated in 50 g γ-sterilised soil in order to reach soil moisture at 60% water-holding capacity. Triplicate microcosms were built up for each of the three diversity levels. Soil microcosms were incubated in the dark at 20°C over a 32-days period. Bacterial communities implantation in γ-sterilised soil was followed by enumeration on nutrient agar (3 g.l^-1^ beef extract, 5 g.l^-1^ peptone, 15 g.l^-1^ agar) supplemented with 100 µg.ml^-1^ cycloheximide (Sigma-Aldrich, Saint-Quentin Fallavier, France) to suppress fungi. Soil samples were collected and kept frozen at -20°C for subsequent DNA extraction and analyses by real time PCR and pyrosequencing. Abundance was PCR quantified by measuring the number of 16S and 18S rDNA fragments per g of soil by using a standard curve approach.

### Bacterial strain

*L. monocytogenes* L9 [37], a spontaneous rifampicin resistant mutant of *L. monocytogenes* strain EGD-e was used in this study. Inocula were prepared by incubating the strain statically at 25°C for 16 h in 5 ml of trypton soy broth (TSB; AES Chemunex, Bruz, France) and subculturing into 10 ml of fresh TSB to an O.D_600nm_ of 0.4.

### Soil invasion assays

Cultures were centrifuged at 8000 g for 5 minutes at room temperature and the pellets were suspended in NaCl (0.85%). Fifty g soil microcosms were inoculated to a final concentration of 2.10^6^ CFU.g^-1^ of soil. Triplicate microcosms were inoculated with three independent inocula. The 9 soil microcosms were then incubated in the dark at 25°C. *L. monocytogenes* L9 populations were enumerated by serial plating on Polymyxin-Acriflavin-Lithium-Chloride-Ceftazidime-Aesculin-Mannitol agar (PALCAM; AES Chemunex, Bruz, France) supplemented with 100 µg.l^-1^ cycloheximide and rifampicin (Sigma-Aldrich, Saint-Quentin Fallavier, France) immediately after inoculation and periodically over a 30-days period.

### DNA extraction

To extract DNA from soil sample, mechanical and chemical lyses with high temperature were used. Two g of soil were added to 8 ml of lysis buffer (tris-HCl 100 mM, EDTA 100 mM, NaCl 100 mM, SDS 2%, ultra pure H_2_O) supplemented with 4 g of silica beads (100 µm), 5 g of ceramic beads (1.4 mm) and 8 glass beads (4 mm). This mixture was shaken in a Fast Prep (MP Bio, Illkirch Graffenstaden, France) during 3 cycles (4 m.s^-1^, 30 seconds) and incubated at 70°C for 30 minutes to disrupt the cells. The sample was then centrifuged at 7 000 g for 5 minutes at room temperature and potassium acetate (3 M, pH 5.5) was added to the supernatant (1:10 v/v). The mixture was incubated on ice for 10 minutes and centrifuged (14 000 g for 5 minutes at 4°C). The aqueous phase was then precipitated with an equal volume of isopropanol (-20°C), washed and dried. The pellet was suspended in 130 µl of ultra pure water and purified once on polyvinyl polypyrrolidone (PVPP; Sigma-Aldrich, Saint-Quentin Fallavier, France) columns and then on Geneclean^®^ columns (MP Bio, Illkirch Graffenstaden, France). DNA concentration was estimated with the Quant-iT dsDNA Assay kit (Invitrogen, Cergy Pontoise, France).

### Pyrosequencing of 16S rDNA genes sequences

Microbial diversity was analysed by 454 pyrosequencing, a molecular technique allowing a rapid and massive production of targeted DNA sequences [38]. A 16S rRNA gene fragment of 440 bases was amplified from DNA extracts using the primers 479F (5’-CAGCMGCYGCNGTAANAC-3’) and 888R (5’-CCGYCAATTCMTTTRAGT-3’) according to the procedure described by Terrat et al [39]. Briefly, for each soil, 5 ng of DNA were used as template into a 25 µl PCR under the following conditions: 94°C for 2 minutes, 35 cycles of 30 seconds at 94°C, 52°C for 30 seconds and 72°C for 1 minute, followed by 7 minutes at 72°C. PCR products were purified using the GenElute^TM^ PCR clean-Up kit (Sigma-Aldrich, Saint-Quentin Fallavier, France) and quantified using the Quant-iT dsDNA Assay kit (Invitrogen, Cergy Pontoise, France). Purified PCR products were specifically tagged in a second PCR of 9 cycles, conducted under similar conditions, with ten base pair multiplex identifiers (MIDs) added to the primers at 5’ position. PCR products were finally purified and quantified as previously described. Pyrosequencing was then carried out on a GS Junior (Roche 454 Sequencing System).

### Analysis of pyrosequencing data using the QIIME pipeline

The generated sequence data were processed by the quantitative insights into microbial ecology (QIIME) pipeline [40]. Briefly, sequences that were less than 300 bp or greater than 500 bp in length, that presented mismatches in the primer sequences, or ambiguous bases were discarded. The remaining sequences were assigned to samples according to their unique MIDs. Chimeric sequences were removed and sequences were clustered into Operational Taxonomic Units (OTUs) (97% sequence similarity) using Usearch quality filter (http://www.drive5.com/usearch/) [41] and the gold. fa reference set. A representative sequence for each OTU was selected based on the most abundant sequence in each OTU. This representative sequence was used for taxonomic identification using the Ribosomal Database Project (RDP) and the reference data sets from Greengenes. Phylogenetic alignment of sequences was done with the PyNAST program with a minimum length of 150 bp and a minimum percent identity of 75.0 [42]. Once the number of sequence reads had been homogenised between microcosms, alpha diversity was used to describe the microbial richness, diversity and evenness within the constructed microcosms using Chao1 (a nonparametric richness estimator based on distribution of singletons and doubletons), Abundance-based Coverage Estimator (ACE; a nonparametric richness estimator based on distribution of abundant (≥10) and rare (<10) OTUs), Shannon, Inverse Simpson and equitability metrics. β-diversity (diversity between groups of samples) was used to test phylogeny-based community composition among the constructed microcosms using weighted Unique Fraction of branches shared (UniFrac) distances [43,44]. The UniFrac distance matrix was analysed by principal coordinate plots (PCoA) and uncertainty in PCoA plots was estimated using jackknife analysis (jackknife replicate = 10). 3D PCoA plots were used to visualize the similarities or dissimilarities of variables that best represent the pair-wise distances between sample groups.

### Statistics

The unilateral Student *t*-test was used to compare richness, diversity and equitability metrics between the undiluted, 10^2^- and 10^4^-diluted microcosms and the analysis of variance (ANOVA) to estimate whether or not differences in relative abundance of bacterial phyla and genera occurred in the different microcosms. A Venn diagram [45] was performed to represent the unique genera detected in the constructed microcosms. Moreover, in order to compare survival pattern of *L. monocytogenes* L9 populations in the constructed microcosms over a 30-days period, the repeated-measures ANOVA was performed. Then, Spearman’s rank correlations was used to test the dependence between the following variables: survival rate, observed OTUs, Chao1, ACE, Shannon, Inverse Simpson and equitability.

## Results

### Microbial abundance evaluation in the constructed microcosms

**Figure 1:**
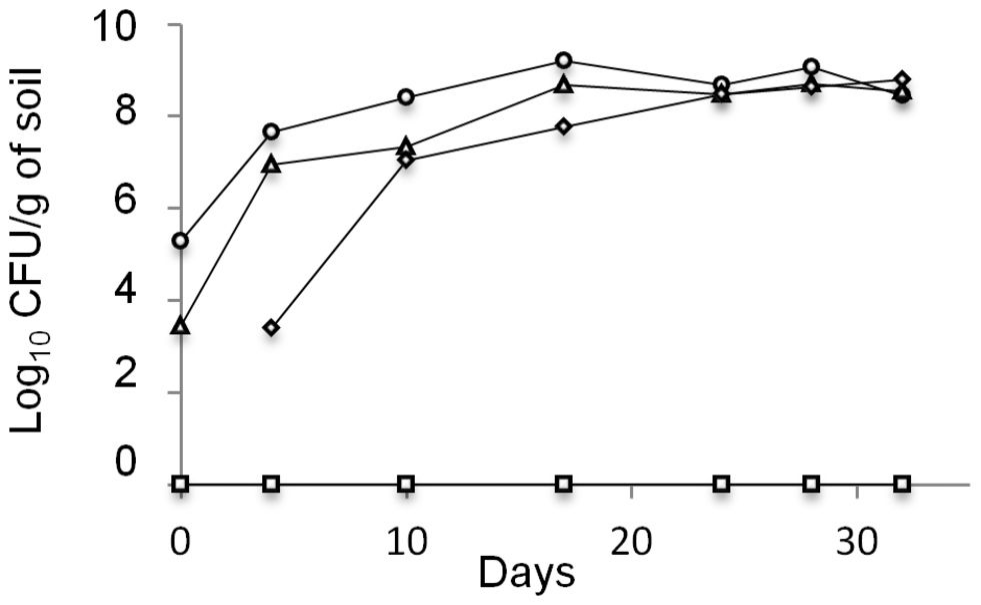
Bacterial communities implantation in γ-sterilised soils over a 32-days period. Undiluted (○), 10^2^- (Δ) and 10^4^-diluted (_◊_) populations and control sterilized soil (□).

**Figure 2:**
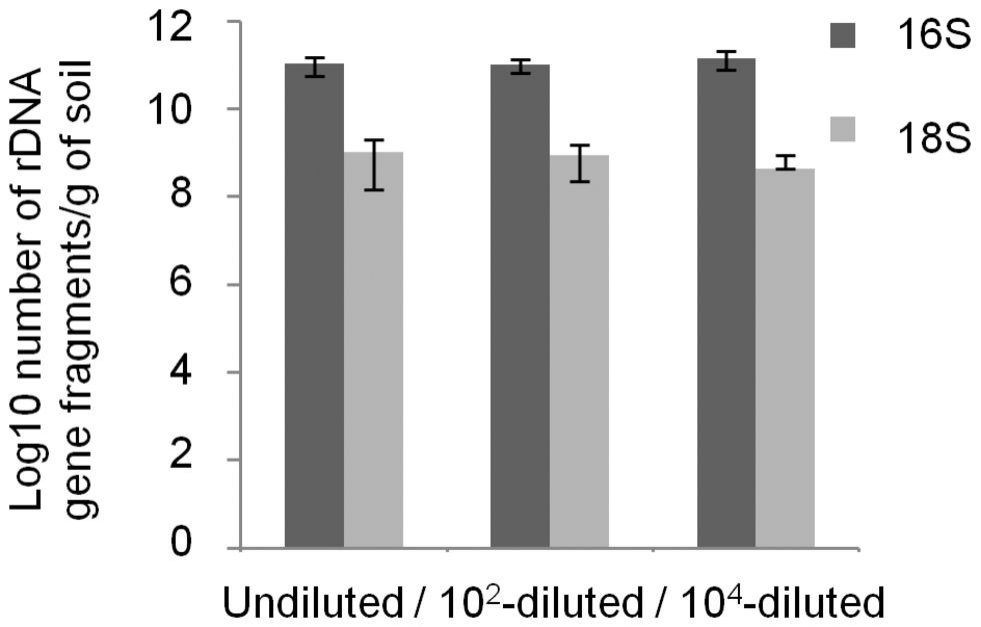
Abundance of 16S and 18S rDNA gene fragments in the constructed microcosms. The number of rDNA gene fragments were determined by real time quantitative PCR from a standard curve relating the quantity of rDNA fragments as a function of the Ct value. The errors bars represent standard deviation from three replicate samples value.

After a period of 32 days to allow colonization of the sterilised soil by the inoculated microorganisms, bacterial abundance did equilibrate in all microcosms (Figure 1). At the end of the equilibration period, populations of culturable microorganisms were stable and similar abundances of approximately 5.10^8^ cells per gram of soil were numerated in all microcosms. In a control γ-irradiated soil microcosm, no culturable microorganisms could be detected over a 62 days period. In agreement with plate counting data, qPCR results showed that all constructed microcosms harboured a similar (student *t*-test, P < 0.05) microbial abundance level (Figure 2).

### Richness, diversity and evenness of the constructed microcosms

We determined the level of bacterial richness, diversity and evenness within samples as a marker of the effectiveness of the dilution-to-extinction approach. OTUs were estimated by the Chao1 metric and by the ACE metric in the undiluted, 10^2^- and 10^4^-diluted microcosms (Table 1). Statistical analyses (unilateral student *t*-test, P < 0.05) indicated that the estimated bacterial species richness was similar in all constructed microcosms. However, contrary to bacterial richness, the Shannon and Inverse Simpson metrics suggested that bacterial diversity differed between microcosms (Table 1) and that diversity was significantly higher in the undiluted microbiota (unilateral student *t*-test, P < 0.05). In spite of the initial 100-fold dilution between the 10^2^- and 10^4^-diluted microbiotas, the differences of bacterial diversity were not significant (unilateral student *t*-test, P < 0.05). Moreover, the representativeness of each OTU followed similar patterns to those observed for diversity metrics (Table 1). Indeed, evenness gradually decreased from the undiluted microbiota, which presented the highest evenness, to the 10^4^-diluted microbiota where evenness was at its lowest level (unilateral student *t*-test, P < 0.05).

**Table 1:** Bacterial richness, diversity and evenness at a genetic distance of 5%.

**Treatment**	**Observed OTUs**	**Chao1**	**ACE**	Shannon**	Inverse Simpson*	Equitability*
Undiluted	927 ± 92	1508 ± 284	1680 ± 326	8.57^a^ ± 0.22	155^a^ ± 34	0.87^a^ ± 0.02
10^2^-diluted	925 ± 132	1849 ± 406	2070 ± 450	8.24**^ab^** ± 0.35	101^b^ ± 30	0.84**^ab^** ± 0.03
10^4^-diluted	939 ± 75	1775 ± 205	1976 ± 260	8.22^b^ ± 0.21	85^b^ ± 23	0.83^b^ ± 0.02

Richness is expressed as the number of observed unique operational units (OTUs) and has been estimated by the estimator Chao1 and the abundance-based coverage estimator (ACE). Diversity is expressed by the Shannon and the Inverse Simpson index. Evenness is measured as the ratio of Shannon index and the number of observed OTUs. Standard deviation was measured from three replicate samples value. Letters indicate values with significant differences after Student test (unilateral *t*-test, *P < 0.05, **P < 0.08).

### Phylogenetic and taxonomic profiles

**Figure 3:**
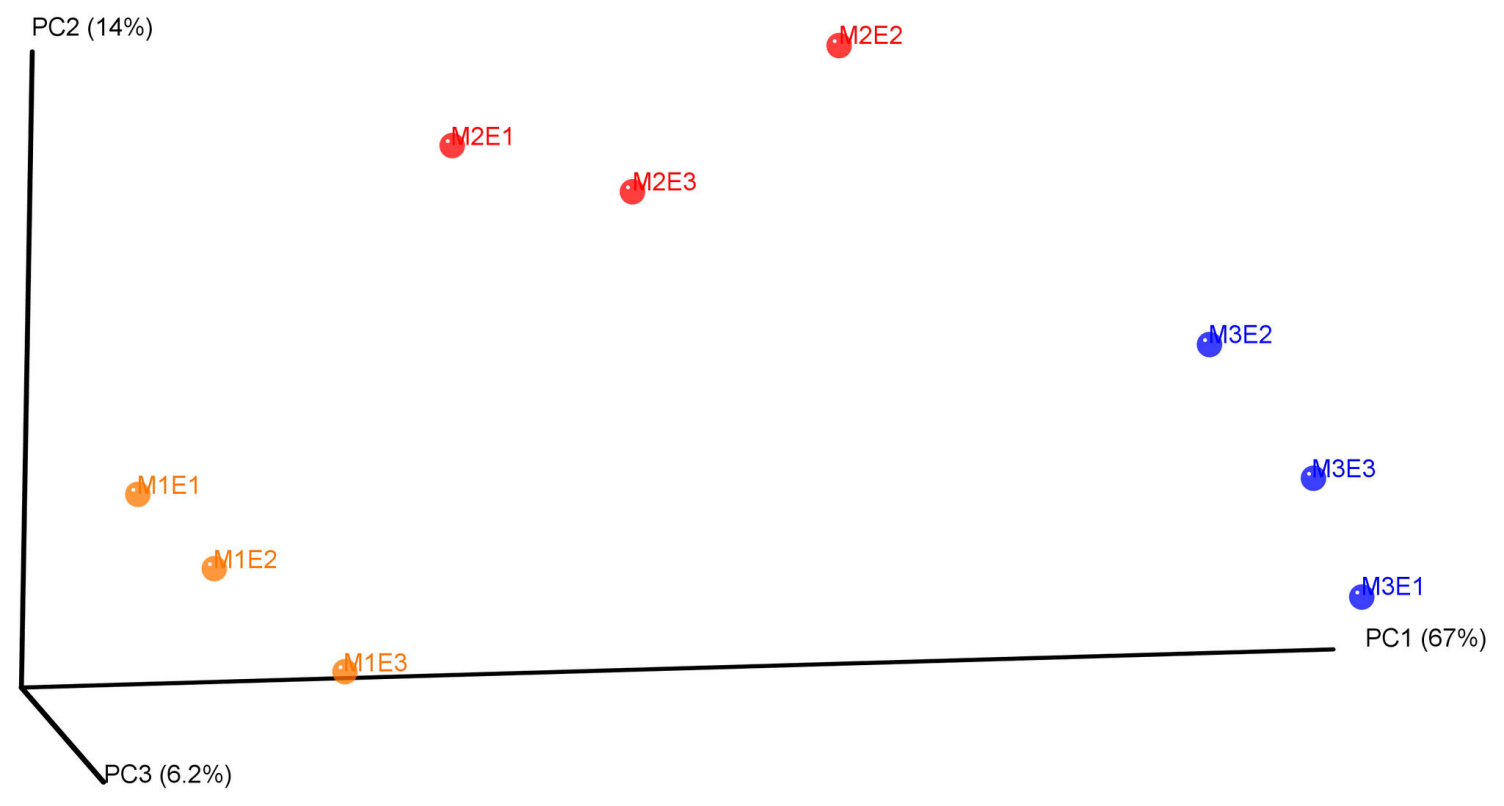
β-diversity analysis of the microcosms composition. Phylogenetic dataset analyzed using jackknifed PCoA of the weighted pairwise UniFrac distances. The undiluted, 10^2^- and 10^4^-diluted microbiotas are respectively represented by orange, red and blue plain circles.

**Figure 4:**
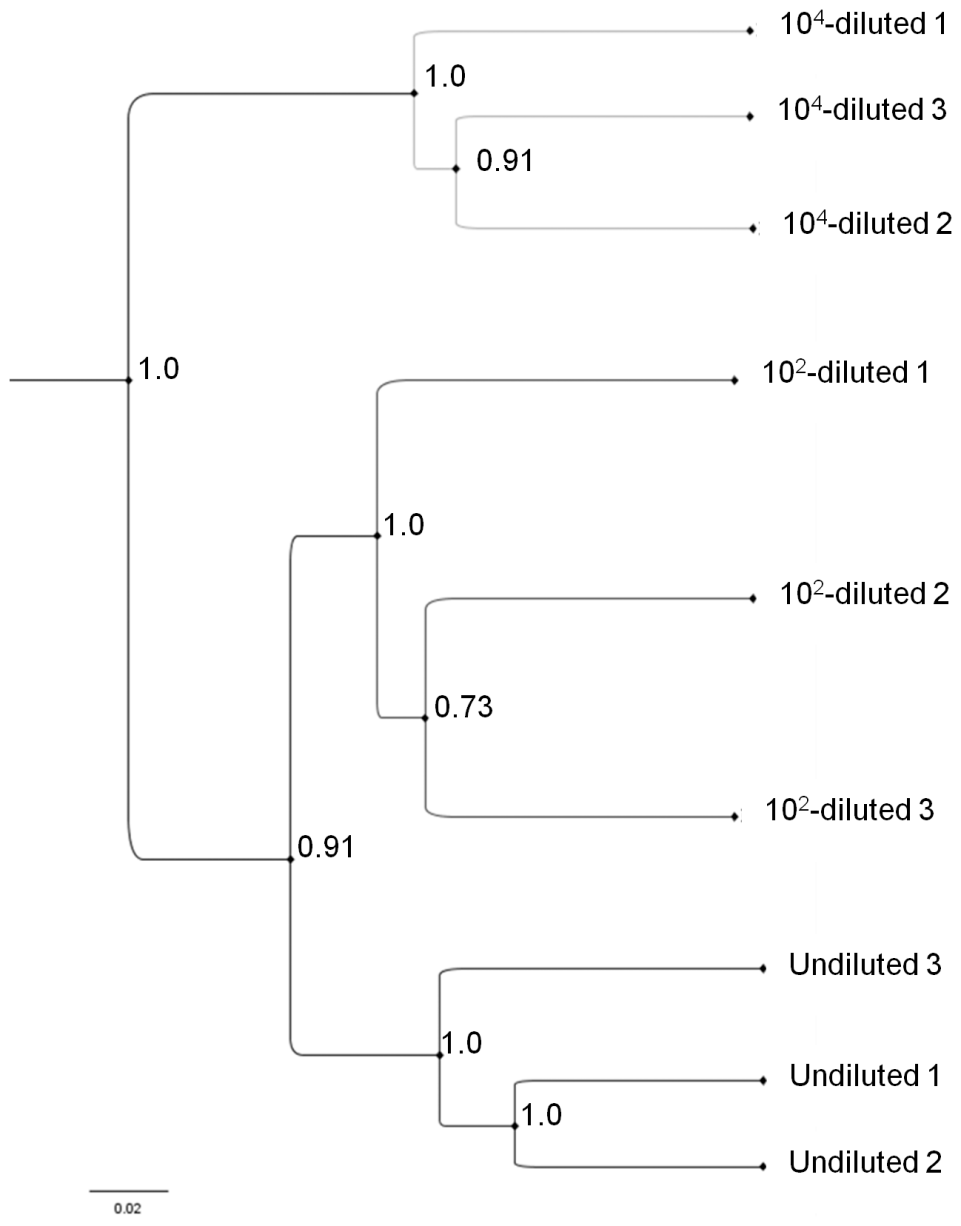
Jackknifed Unweighted Pair Group Method with Arithmetic mean (UPGMA) cluster tree of the constructed microcosms. UPGMA clustering was made from the 10 jackknifed weighted UniFrac distance matrix generated for each constructed microcosm.

Differences were further characterised by comparing phylogenetic and taxonomic profiles. The 3D PCoA plots visualization (Figure 3) explained over 87% of the variation of microbiotas. Principal coordinate 1 explained 67% of the variation in the data and distinguished the three diversity levels, with undiluted and 10^4^-diluted microbiota being strongly discriminated and 10^2^-diluted microbiota represented an intermediate situation. According to the UniFrac tree-based metric, phylogenetic profiles of the three constructed microbiotas were significantly different. Moreover the UPGMA cluster tree also clustered the constructed microcosms according to the dilution treatment (Figure 4). It also indicates that phylogenetic profile of the undiluted microbiota is closer to the 10^2^-diluted than the 10^4^-diluted phylogenetic profile.

From all sequences that were assigned to the domain Bacteria, a total of 21 phyla were detected (Table 2). Among the abundant phyla (>1% of total reads), the relative abundance of the *Proteobacteria*, *Actinobacteria* and *Firmicutes* significantly diverged depending on the diversity level (ANOVA, tukey test, P < 0.05). Indeed, the relative abundance of *Proteobacteria* significantly decreased over the dilution treatment. On the contrary, the relative abundance of *Actinobacteria* significantly increased from 25% in the undiluted microcosm to 42% and 40% in the 10^2^- and 10^4^-diluted microcosms, respectively. The relative abundance of *Firmicutes* was significantly higher in the 10^4^-diluted microcosms (28%) but the differences between the undiluted and 10^2^-diluted were not statistically significant. Moreover, the *Planctomycetes* decreased down to extinction in the 10^4^-diluted. Finally, similar abundance of *Bacteroidetes* was detected in all microcosms.

**Table 2:** Phylogenetic communities composition of microcosms.

**Phylum**	**Undiluted**	**10^2^-diluted**	**10^4^-diluted**
*Proteobacteria*	56.2^a^ ± 4.4	37.0^b^ ± 7.7	27.5^c^ ± 2.5
*Actinobacteria*	25.2^a^ ± 0.5	41.7^b^ ± 4.2	39.5^b^ ± 3.3
*Firmicutes*	8.8^ab^ ± 4.0	16.0^b^ ± 4.7	27.7^c^ ± 1.4
*Planctomycetes*	1.4 ± 0.2	0.3 ± 0.1	0.0 ± 0.0
*Bacteroidetes*	3.6 ± 0.6	1.4 ± 0.7	0.6 ± 0.9
Others	1.9 ± 0.4	2.5 ± 1.0	3.0 ± 0.5

Relative abundance (%) of detected phyla. Phylogenetic groups accounting for less than 1% of all classified sequences are summarized in the artificial group “others”. Standard deviation was measured from three replicate samples value. Letters indicate significant differences of relative abundance between microcosms after ANOVA (Tukey test, P < 0.05).

**Figure 5:**
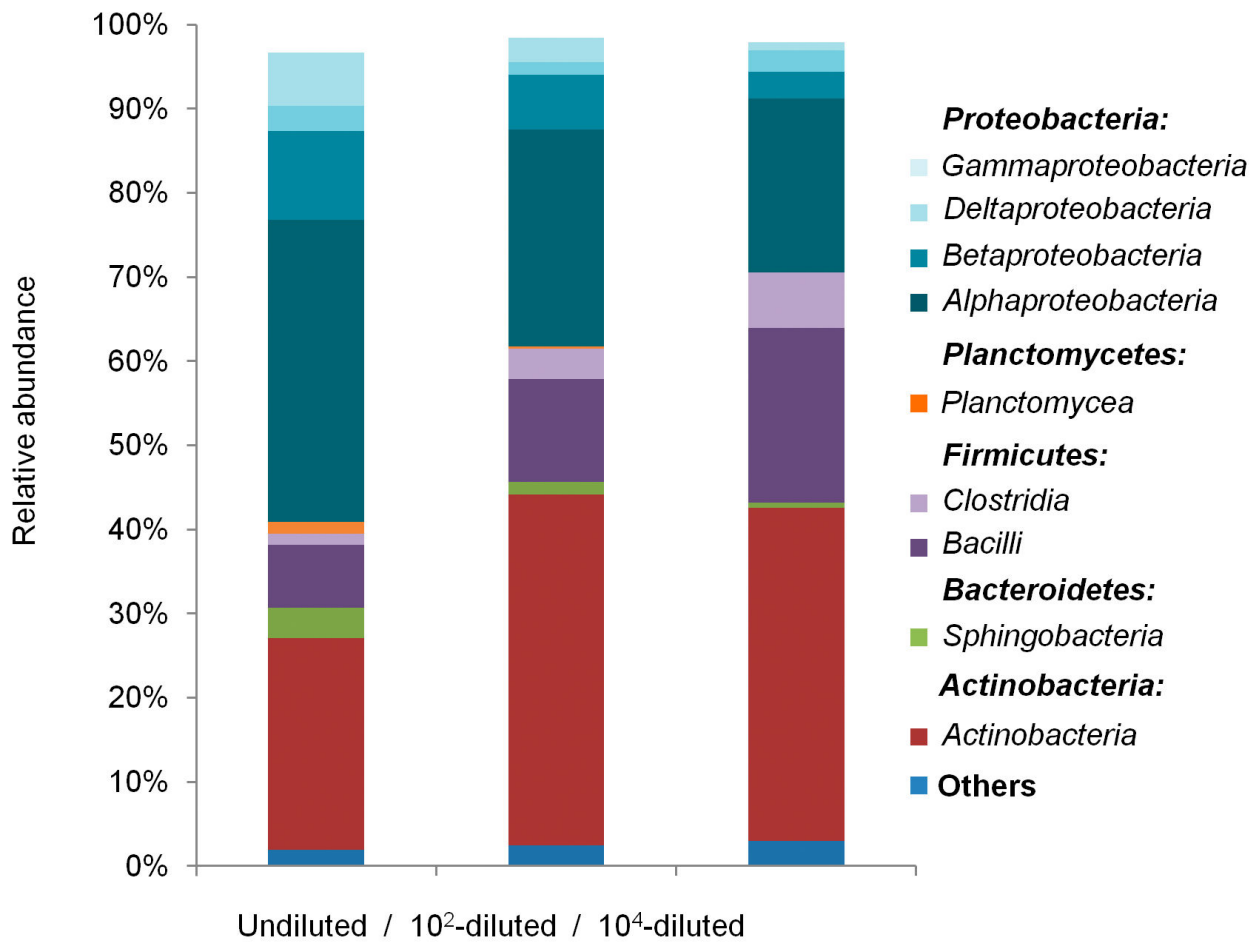
Phylogenetic community composition of microcosms. Relative abundance (%) of detected classes.

To complete the characterization of the constructed microcosms, taxonomic assignment was done at the class and genus levels. Fifty three classes were detected from the assigned sequences, of which 10 classes were abundant (Figure 5). *Alphaproteobacteria*, *Betaproteobacteria*, *Deltaproteobacteria* and *Gammaproteobacteria* were detected in all microcosms and *Alphaproteobacteria* and *Betaproteobacteria* alone accounted for over 84% of the phylum. In all microcosms, the phylum *Firmicutes* was represented by the *Clostridia* and *Bacilli*. The relative abundance of *Clostridia* did not vary significantly while *Bacilli* were more abundant in the 10^4^-diluted microcosms compared to the undiluted and 10^2^-diluted microcosms that were not statistically different (ANOVA, Tukey, P < 0.05). *Planctomycea*, *Sphingobacteria* and *Actinobacteria* respectively were the only classes detected for the phyla *Planctomycetes*, *Bacteroidetes* and *Actinobacteria*. Concerning genus taxonomic assignment, 370 genera were detected, 38 as abundant (> 1% of total reads) and 332 as rare genera (

**Figure 6:**
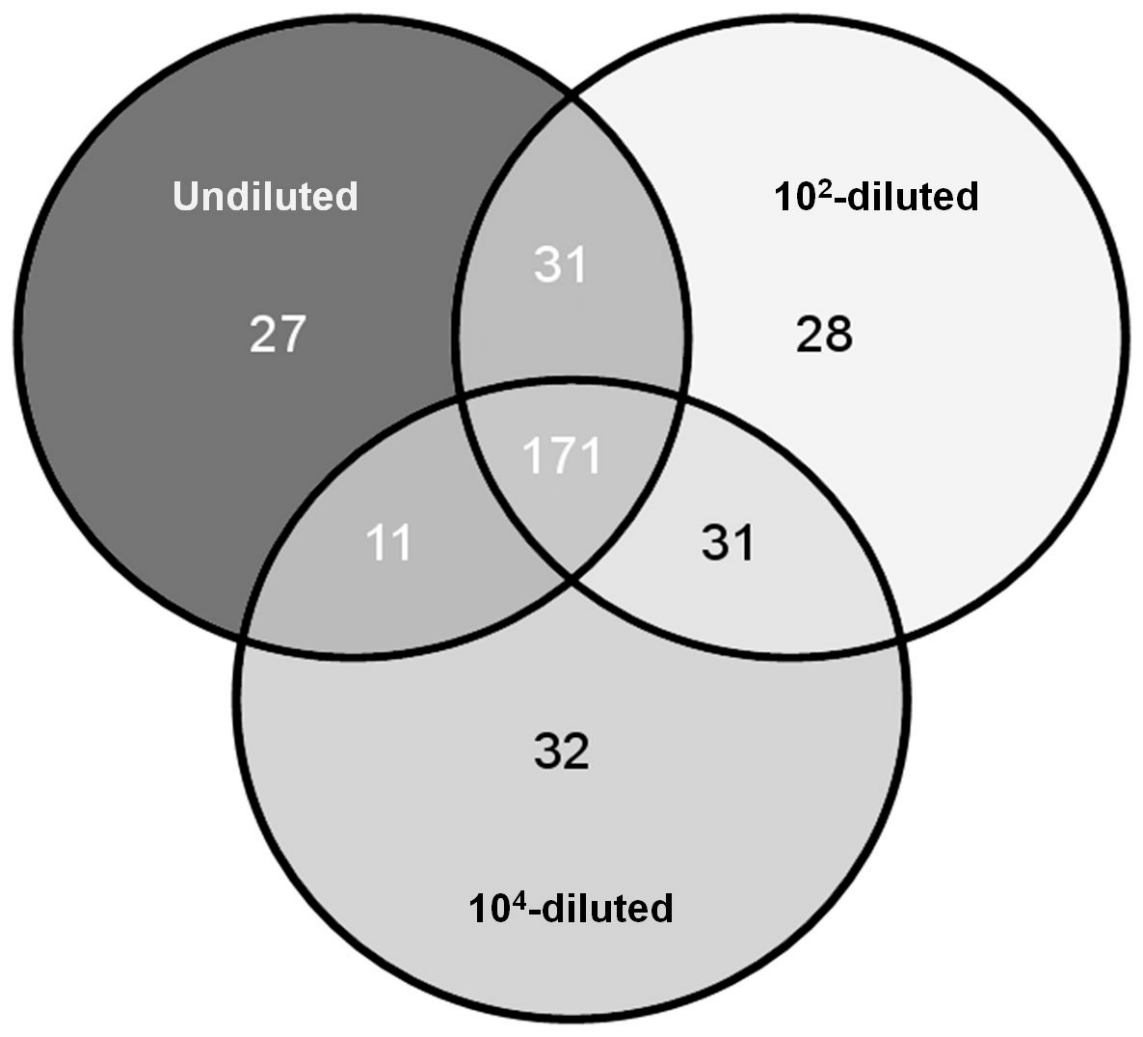
Venn diagram representing shared and unique rare genera (< 1% of total reads) of microcosms.

< 1% of total reads). Among the abundant genera, the relative abundance of 6 of them significantly differed among the constructed microcosms (Table 3). In addition, microcosms also differed in terms of rare genera (Table S1). Among the 332 rare genera, 27, 28 and 32 specific genera were respectively detected in the undiluted, 10^2^- and 10^4^-diluted microcosms and 31, 31 and 11 rare genera were respectively shared between the undiluted and 10^2^-diluted, 10^2^- and 10^4^-diluted and the undiluted and 10^4^-diluted microcosms (Figure 6).

**Table 3:** Relative abundance (%) of abundant genera (> 1% of total reads) detected as significantly different between microcosms.

**Phylum**	**Class**	**Genus**	**Undiluted**	**10^2^-diluted**	**10^4^-diluted**
*Actinobacteria*	*Actinobacteria*	Unknown member of the 0319-7L14	0.4^a^ ± 0.1	4.0^a^ ± 3.3	13.4^b^ ± 2.3
*Actinobacteria*	*Actinobacteria*	*Kribella*	4.2^a^ ± 1.3	6.4^a^ ± 3.4	1.1^b^ ± 0.5
*Actinobacteria*	*Actinobacteria*	Other member of the *Streptomycetaceae* *	3.1^a^ ± 0.8	7.8^b^ ± 3.1	3.9^ab^ ± 2.6
*Actinobacteria*	*Actinobacteria*	*Streptomyces*	4.4^ab^ ± 0.6	7.7^a^ ± 4	3.2^b^ ± 1.2
*Firmicutes*	*Bacilli*	Other member of the *Bacillales* **	4.3^a^ ± 3.1	7.3^a^ ± 2.4	14.5^b^ ± 1.6
*Proteobacteria*	*Betaproteobacteria*	Other member of the *Comamonadaceae* ***	5.8^a^ ± 3.4	4.6^ab^ ± 3.5	1.3^b^ ± 1.3

* Other member of the *Streptomycetaceae*: other than *Kitasatospora* and *Streptomyces*.

** Other member of the *Bacillales*: other than *Alicyclobacillus*, *Bacillus*, *Geobacillus*, *Ammoniphilus*, *Brevibacillus*, *Cohnella*, *Paenibacillus*, *Kurthia*, *Paenisporosarcina*, *Rummeliibacillus*, *Solibacillus*, *Sporosarcina*, *Ureibacillus* and *Viridibacillus*.

*** Other member of the *Comamonadaceae*: other than *Polaromonas*, *Variovorax* and *Xenophilus*.

Standard deviation was measured from three replicate samples value. Letters indicate values with significant differences after ANOVA (Tukey test, P < 0.05).

As expected, phylogenic and taxonomic data confirmed that the dilution-to-extinction method generated significant dissimilarities between microcosms at the phylum, class and genus levels.

### Invasion of the pathogen

**Figure 7:**
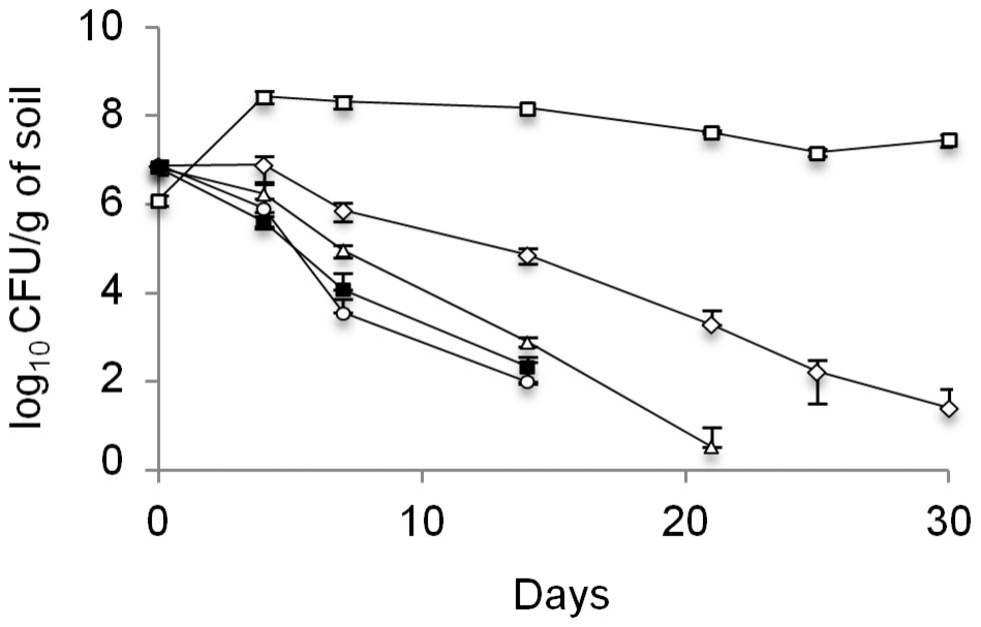
*L. monocytogenes* L9 survival over a 30-days period. In a natural soil (■), in the constructed soil microcosms with established undiluted (○), 10^2^- (Δ) and 10^4^-diluted (_◊_) microbiotas and in a γ-sterilised soil (□). The errors bars represent standard deviation from three replicate samples value.

In natural soil microcosms, a progressive decline of *L. monocytogenes* L9 was observed (Figure 7). Over 5 log decrease was recorded during the first fourteen days of incubation and the population was no longer detected after this period. A similar pattern was observed in the undiluted microcosms. A decline was also observed in the 10^2^-diluted microcosms but the decrease was significantly lesser (ANOVA, P < 0.05) than in the natural and undiluted microcosms, with only 4 log decrease observed 14 days after inoculation. Moreover, populations remained detectable up to day 21. In the 10^4^-diluted microcosms, populations were stable during the first four days of incubation. After this period, a decrease was observed and the relative abundance of *L. monocytogenes* L9 remained significantly higher (ANOVA, P < 0.05) throughout the duration of the experiment. Indeed, 2 log and 4 log decreases were respectively recorded at days 14 and 21. Furthermore, populations were still detected up to the end of the experiment. Finally, in the absence of microflora, *L. monocytogenes* L9 populations increased of over 2 log within the first four days of incubation, and the relative abundance of the population was stable until the end of the experiment.

The Spearman rank correlation test was used to check correlations between the survival rate of *L. monocytogenes* L9 and the richness, diversity and evenness metrics of the constructed microcosms. The dependence of the survival rate was tested with the observed OTUs, Chao1, ACE, Shannon, Inverse Simpson and equitability. From these six metrics, a significant negative correlation was detected between the survival rate and the Inverse Simpson (*ρ*_s_ = -0.817, P < 0.05) and also between the survival rate and the evenness metric (*ρ*_s_ = -0.717, P < 0.05).

## Discussion

Although *L. monocytogenes* is considered as a telluric bacterium, its ecology in soil is poorly understood. In this study, we investigated whether microbial species diversity is a major driver of the ecosystem service of control of pathogenic organisms. We experimentally eroded soil microbial diversity and characterised the induced differences by high throughput pyrosequencing of bacterial 16S rDNA. The erosion process generated diversity and evenness gradients. As expected, the highest differences were observed between the undiluted and the 10^4^-diluted microcosms while it was intermediate in the 10^2^-diluted microcosms. Taxonomic assignments further evidenced these changes. Among the 21 phyla detected, a third was detected as abundant. The erosion process resulted in a significant decrease of the relative abundance of *Proteobacteria* and members of the *Planctomycetes* were eventually not recovered. Conversely the relative abundance of *Actinomycetes* and *Firmicutes* increased in the most eroded microcosms. These differences could be assigned to the variation of the relative abundance of six genera identified as abundant. Investigation of the phylogenetic assignment of rare genera did illustrate further dissimilarities between microcosms. Indeed, unique groups were detected within each microcosm. These alterations of the phylogenetic composition and diversity are consistent with results of other studies manipulating diversity in microbial systems [46-49]. These studies showed that the structure, diversity, functional traits or stability of the communities were affected by dilution.

*L. monocytogenes* invasion was significantly lower in the most diverse, undiluted microcosms. This result provides evidence that highly diverse soil microbial communities may act as a barrier against *L. monocytogenes* invasion. This was supported by Spearman’s rank correlations test which showed a negative correlation between the level of diversity and the survival rate of *L. monocytogenes* in soil microcosms. These results demonstrate, at a microbial scale, that diversity is a major provider of the biological barrier of ecosystems against biological invasion. This is in accordance with the current ecological theory of biological invasion that highlights the correlation between the degree of diversity and the protection of the ecosystem from invasive species [26].

Moreover, another dimension of microbial community composition has to be considered. Indeed, the barrier effect against *L. monocytogenes* invasion was lesser in the 10^4^-diluted than in the 10^2^-diluted constructed microcosms although bacterial diversity and evenness were similar. However, analyses at the genus level showed significant differences between these microcosms, suggesting that the actual phylogenetic composition of the indigenous community may also contribute to the barrier effect. Recent reports showed that phylogenetically diverse soil communities favour soil ecosystem stability and functioning [50-53]. Similarly, assembly of complementary plant and animal species increases functioning and protection against invasion [54,55]. As a rule, components of microbial ecosystems engage in complex interactions that regulate microbial communities [56]. From our results, one can assume that at a same level of species diversity, complementary species and interactions within the complex soil communities can direct the fate of invading microorganisms.

Overall, this study highlights the critical role that microbial diversity and structure play in the biological barrier effect of soil against invasion by pathogenic bacteria. This suggests that, in the environments, soil diversity and phylogenetic composition may impact the fate and circulation of pathogenic microorganisms and thus the overall associated health hazard. Moreover, this study confirms that the body of ecological theory developed to explain biological invasion by plants and animals does apply to microorganisms. As demonstrated in this study, microbial models are useful to address experimentally ecology theories and concepts while the understanding of the ecology of microorganisms requires the input of ecological theory [25]. Further work is required in order to address the relative weight of the degree of diversity versus the phylogenetic composition of soil microbiota in the biological barrier that these microorganisms promote against invasion by pathogenic bacteria.

## Supporting Information

Table S1 **Relative abundance of rare genera detected in the constructed microcosms.**
(DOCX)
