# Supplementary material for: Microbial Diversity and Structure Are Drivers of the Biological Barrier Effect against Listeria monocytogenes in Soil
Source: PLoS One. 2013 Oct 8;8(10):e76991. doi: 10.1371/journal.pone.0076991 (PMC3792895; doi:10.1371/journal.pone.0076991)
Supplement: Table S1 — Relative abundance of rare genera detected in the constructed microcosms. (DOCX) [file pone.0076991.s001.docx]

Table S1. Relative abundance of rare genera detected in the constructed microcosms.

| **Phylum; Class (c); Order (o); Family (f); Genus (g)** | **Un-diluted** | **10^2^-diluted** | **10^4^-diluted** |
| --- | --- | --- | --- |
| **Unique rare genera of the undiluted microcosms** |  |  |  |
| *Acidobacteria;c Acidobacteria;o Acidobacteriales;f Acidobacteriaceae*;Other | 0,009% | 0,000% | 0,000% |
| *Acidobacteria;c Chloracidobacteria;o ;f ;g* | 0,032% | 0,000% | 0,000% |
| *Actinobacteria;c Actinobacteria;o Acidimicrobiales;f Iamiaceae*;Other | 0,009% | 0,000% | 0,000% |
| *Actinobacteria;c Actinobacteria;o Actinomycetales;f Pseudonocardiaceae;g* | 0,019% | 0,000% | 0,000% |
| *Armatimonadetes;c S1a-1H;o ;f ;g* | 0,009% | 0,000% | 0,000% |
| *Bacteroidetes;c Sphingobacteria;o Sphingobacteriales*;Other;Other | 0,027% | 0,000% | 0,000% |
| *Bacteroidetes;c Sphingobacteria;o Sphingobacteriales;f Flexibacteraceae;g Emticicia* | 0,009% | 0,000% | 0,000% |
| *Bacteroidetes;c Sphingobacteria;o Sphingobacteriales;f Flexibacteraceae;g Spirosoma* | 0,013% | 0,000% | 0,000% |
| *Chloroflexi;c Anaerolineae*;Other;Other;Other | 0,009% | 0,000% | 0,000% |
| *Chloroflexi;c Anaerolineae*;o A4b;f ;g | 0,029% | 0,000% | 0,000% |
| *Chloroflexi;c Anaerolineae;o Anaerolineales;f Anaerolinaceae;g Anaerolinea* | 0,035% | 0,000% | 0,000% |
| *Chloroflexi;c Anaerolineae*;o GCA004;f ;g | 0,014% | 0,000% | 0,000% |
| *Chloroflexi*;c Bljii12;o AKYG885;f Dolo 23;g | 0,040% | 0,000% | 0,000% |
| *Chloroflexi;c Chloroflexi;o Roseiflexales;f Kouleothrixaceae*;g | 0,120% | 0,000% | 0,000% |
| *Firmicutes;c Bacilli;o Bacillales;f Planococcaceae;g Viridibacillus* | 0,018% | 0,000% | 0,000% |
| *Gemmatimonadetes;c Gemmatimonadetes;o Gemmatimonadales;f Gemmatimonadaceae;g* | 0,047% | 0,000% | 0,000% |
| *Gemmatimonadetes;c Gemmatimonadetes;o Gemmatimonadales;f Gemmatimonadaceae;g Gemmatimonas* | 0,009% | 0,000% | 0,000% |
| *Nitrospirae;c Nitrospira;o Nitrospirales*;f FW;g 4-29 | 0,029% | 0,000% | 0,000% |
| *Planctomycetes*;c agg27;o CL500-15;f ;g | 0,019% | 0,000% | 0,000% |
| *Proteobacteria;c Betaproteobacteria;o Burkholderiales;f Burkholderiaceae*;Other | 0,040% | 0,000% | 0,000% |
| *Proteobacteria;c Betaproteobacteria;o Nitrosomonadales;f Nitrosomonadaceae*;Other | 0,009% | 0,000% | 0,000% |
| *Proteobacteria;c Betaproteobacteria;o Nitrosomonadales;f Nitrosomonadaceae;g Nitrosomonas* | 0,329% | 0,000% | 0,000% |
| *Proteobacteria;c Betaproteobacteria;o Rhodocyclales;f Rhodocyclaceae;g Dechloromonas* | 0,019% | 0,000% | 0,000% |
| *Proteobacteria;c Deltaproteobacteria;o Entotheonellales;f Entotheonellaceae;g CandidatusEntotheonella* | 0,023% | 0,000% | 0,000% |
| *Proteobacteria;c Gammaproteobacteria;o Xanthomonadales;f Xanthomonadaceae;g Stenotrophomonas* | 0,036% | 0,000% | 0,000% |
| *Proteobacteria;c Gammaproteobacteria;o Xanthomonadales;f Xanthomonadaceae;g Xanthomonas* | 0,009% | 0,000% | 0,000% |
| WS3;c PRR-12;o Sediment-1;f PRR-10;g | 0,009% | 0,000% | 0,000% |
| **Unique rare genera of the 10^2^-diluted microcosms** |  |  |  |
| *Acidobacteria;*c RB25;o ;f ;g | 0,000% | 0,003% | 0,000% |
| *Actinobacteria;c Actinobacteria;o Actinomycetales;f* *Actinosynnemataceae*;Other | 0,000% | 0,038% | 0,000% |
| *Actinobacteria;c Actinobacteria;o Actinomycetales;f Actinosynnemataceae;g Lentzea* | 0,000% | 0,003% | 0,000% |
| *Actinobacteria;c Actinobacteria;o Actinomycetales;f Cryptosporangiaceae;g* | 0,000% | 0,003% | 0,000% |
| *Actinobacteria;c Actinobacteria;o Actinomycetales;f Intrasporangiaceae;g* | 0,000% | 0,009% | 0,000% |
| *Actinobacteria;c Actinobacteria;o Actinomycetales;f Intrasporangiaceae;g Knoellia* | 0,000% | 0,007% | 0,000% |
| *Actinobacteria;c Actinobacteria;o Actinomycetales;f Micromonosporaceae;g Catellatospora* | 0,000% | 0,005% | 0,000% |
| *Actinobacteria;c Actinobacteria;o Actinomycetales;f Nocardiaceae;g Skermania* | 0,000% | 0,003% | 0,000% |
| *Actinobacteria;c Actinobacteria;o Actinomycetales;f Pseudonocardiaceae;g Actinomycetospora* | 0,000% | 0,003% | 0,000% |
| *Actinobacteria;c Actinobacteria;o Actinomycetales;f Thermomonosporaceae*;Other | 0,000% | 0,016% | 0,000% |
| *Bacteroidetes;c Flavobacteria;o Flavobacteriales;f Cryomorphaceae;g* | 0,000% | 0,005% | 0,000% |
| *Chlorobi*;c SM1B09;o ;f ;g | 0,000% | 0,008% | 0,000% |
| *Chloroflexi*;Other;Other;Other;Other | 0,000% | 0,006% | 0,000% |
| *Chloroflexi*;c RA13C7;o ;f ;g | 0,000% | 0,008% | 0,000% |
| *Firmicutes;c Bacilli;o Bacillales;f Paenibacillaceae;g* | 0,000% | 0,010% | 0,000% |
| *Firmicutes;c Bacilli;o Bacillales;f Thermoactinomycetaceae;g* | 0,000% | 0,008% | 0,000% |
| *Firmicutes;c Bacilli;o Bacillales;f Thermoactinomycetaceae;g Mechercharimyces* | 0,000% | 0,003% | 0,000% |
| *Firmicutes;c Clostridia;o Clostridiales;f ClostridialesFamilyXIII,IncertaeSedis;g Eubacterium* | 0,000% | 0,021% | 0,000% |
| *Firmicutes;c Clostridia;o Clostridiales;f Eubacteriaceae;g* | 0,000% | 0,005% | 0,000% |
| *Firmicutes;c Clostridia;o Clostridiales;f Peptococcaceae;g Dehalobacter* | 0,000% | 0,003% | 0,000% |
| *Proteobacteria;c Alphaproteobacteria;o Caulobacterales;f Caulobacteraceae;g* | 0,000% | 0,006% | 0,000% |
| *Proteobacteria;c Alphaproteobacteria;o Rhizobiales;f Rhizobiaceae;g* | 0,000% | 0,005% | 0,000% |
| *Proteobacteria;c Alphaproteobacteria;o Rhizobiales;f Xanthobacteraceae*;Other | 0,000% | 0,003% | 0,000% |
| *Proteobacteria;c Alphaproteobacteria;o Rhodospirillales;f Acetobacteraceae;g Roseomonas* | 0,000% | 0,009% | 0,000% |
| *Proteobacteria;c Gammaproteobacteria;o Chromatiales;f* ;Other | 0,000% | 0,008% | 0,000% |
| *Proteobacteria;c Gammaproteobacteria;o Legionellales;f Coxiellaceae;g Rickettsiella* | 0,000% | 0,003% | 0,000% |
| *Proteobacteria;c Gammaproteobacteria;o Pseudomonadales;f Moraxellaceae*;Other | 0,000% | 0,003% | 0,000% |
| WS3;c PRR-12;o ;f ;g | 0,000% | 0,008% | 0,000% |
| **Unique rare genera of the 10^4^-diluted microcosms** |  |  |  |
| *Acidobacteria*;c iii1-8;o SJA-36;f ;g | 0,000% | 0,000% | 0,011% |
| *Actinobacteria;c Actinobacteria;o Actinomycetales;f Catenulisporaceae;g Catenulispora* | 0,000% | 0,000% | 0,007% |
| *Actinobacteria;c Actinobacteria;o Actinomycetales;f Frankiaceae;g* | 0,000% | 0,000% | 0,008% |
| *Actinobacteria;c Actinobacteria;o Actinomycetales;f Microbacteriaceae;g* | 0,000% | 0,000% | 0,010% |
| *Actinobacteria;c Actinobacteria;o Actinomycetales;f Propionibacteriaceae*;Other | 0,000% | 0,000% | 0,006% |
| *Actinobacteria;c Actinobacteria;o Actinomycetales;f Thermomonosporaceae;g Actinoallomurus* | 0,000% | 0,000% | 0,008% |
| *Actinobacteria;c Actinobacteria;o Actinomycetales;f Thermomonosporaceae;g Actinocorallia* | 0,000% | 0,000% | 0,008% |
| *Actinobacteria;c Actinobacteria;o Rubrobacterales;f Rubrobacteraceae;g* | 0,000% | 0,000% | 0,016% |
| *Actinobacteria;c Actinobacteria;o Solirubrobacterales;f Solirubrobacteraceae*;Other | 0,000% | 0,000% | 0,011% |
| *Bacteroidetes;c Sphingobacteria;o Sphingobacteriales;f Sphingobacteriaceae*;Other | 0,000% | 0,000% | 0,007% |
| *Chloroflexi;c Anaerolineae;o Anaerolineales;f Anaerolinaceae*;Other | 0,000% | 0,000% | 0,015% |
| *Chloroflexi;c Chloroflexi;o Herpetosiphonales;f Herpetosiphonaceae;g Herpetosiphon* | 0,000% | 0,000% | 0,106% |
| *Crenarchaeota;c Thaumarchaeota*;Other;Other;Other | 0,000% | 0,000% | 0,009% |
| *Firmicutes;c Clostridia;o Clostridiales;f ;g* | 0,000% | 0,000% | 0,006% |
| *Firmicutes;c Clostridia;o Clostridiales;f ClostridialesFamilyXI,IncertaeSedis;g Soehngenia* | 0,000% | 0,000% | 0,006% |
| *Firmicutes;c Clostridia;o Clostridiales;f Lachnospiraceae;g Ruminococcus* | 0,000% | 0,000% | 0,006% |
| *Firmicutes;c Clostridia;o Clostridiales;f Symbiobacteriaceae;g* | 0,000% | 0,000% | 0,015% |
| *Firmicutes;c Clostridia;o Halanaerobiales;f Halanaerobiaceae*;Other | 0,000% | 0,000% | 0,008% |
| *Nitrospirae;c Nitrospira;o Nitrospirales;f Thermodesulfovibrionaceae*;Other | 0,000% | 0,000% | 0,006% |
| *Planctomycetes;c Planctomycea;o Gemmatales;f Gemmataceae;g* | 0,000% | 0,000% | 0,006% |
| *Proteobacteria;c Alphaproteobacteria;o Rhizobiales;f Beijerinckiaceae;g Beijerinckia* | 0,000% | 0,000% | 0,027% |
| *Proteobacteria;c Deltaproteobacteria;o Desulfuromonadales;f Desulfuromonadaceae;g Desulfuromonas* | 0,000% | 0,000% | 0,009% |
| *Proteobacteria;c Deltaproteobacteria;o Myxococcales;f Polyangiaceae;Other* | 0,000% | 0,000% | 0,021% |
| *Proteobacteria;c Deltaproteobacteria;o Myxococcales;f Polyangiaceae;g Sorangium* | 0,000% | 0,000% | 0,008% |
| *Proteobacteria;c Gammaproteobacteria;o Legionellales;f Coxiellaceae;g* | 0,000% | 0,000% | 0,010% |
| *Proteobacteria;c Gammaproteobacteria;o Oceanospirillales;f HTCC2089;g* | 0,000% | 0,000% | 0,007% |
| *Proteobacteria;c Gammaproteobacteria;o Xanthomonadales;f Sinobacteraceae;g Hydrocarboniphaga* | 0,000% | 0,000% | 0,007% |
| *Spirochaetes;c Spirochaetes;o Spirochaetales;f Spirochaetaceae*;Other | 0,000% | 0,000% | 0,007% |
| *Tenericutes;c Erysipelotrichi;o Erysipelotrichales;f Erysipelotrichaceae;g Clostridium* | 0,000% | 0,000% | 0,007% |
| *Tenericutes;c Mollicutes;o Entomoplasmatales;f Entomoplasmataceae*;Other | 0,000% | 0,000% | 0,007% |
| *Tenericutes;c Mollicutes*;o RF39;f ;g | 0,000% | 0,000% | 0,006% |
| WS3;c PRR-12;o Sediment-1;f ;g | 0,000% | 0,000% | 0,006% |
| **Rare genera shared between the undiluted and the 10^2^-diluted microcosms** |  |  |  |
| *Acidobacteria*;Other;Other;Other;Other | 0,004% | 0,004% | 0,000% |
| *Actinobacteria;c Actinobacteria;o Actinomycetales;f Micromonosporaceae;g Verrucosispora* | 0,075% | 0,022% | 0,000% |
| *Actinobacteria;c Actinobacteria;o Actinomycetales;f Nocardioidaceae;g Aeromicrobium* | 0,023% | 0,008% | 0,000% |
| *Actinobacteria;c Actinobacteria;o Actinomycetales;f Streptosporangiaceae;g* | 0,031% | 0,037% | 0,000% |
| *Bacteroidetes*;Other;Other;Other;Other | 0,009% | 0,040% | 0,000% |
| *Bacteroidetes;c Flavobacteria;o Flavobacteriales;f Flavobacteriaceae*;Other | 0,004% | 0,006% | 0,000% |
| *Bacteroidetes;c Sphingobacteria;o Sphingobacteriales;f ;g Niastella* | 0,084% | 0,029% | 0,000% |
| *Bacteroidetes;c Sphingobacteria;o Sphingobacteriales;f Flexibacteraceae;g Dyadobacter* | 0,256% | 0,090% | 0,000% |
| *Bacteroidetes;c Sphingobacteria;o Sphingobacteriales;f Sphingobacteriaceae;g* | 0,148% | 0,010% | 0,000% |
| *Bacteroidetes;c Sphingobacteria;o Sphingobacteriales;f Sphingobacteriaceae;g Sphingobacterium* | 0,009% | 0,014% | 0,000% |
| *Chloroflexi;c Thermomicrobia;o Thermomicrobiales;f ;g* | 0,019% | 0,002% | 0,000% |
| *Planctomycetes;c Planctomycea;o Gemmatales;f Gemmataceae;g Gemmata* | 0,004% | 0,008% | 0,000% |
| *Planctomycetes;c Planctomycea;o Gemmatales;f Isosphaeraceae;g* | 0,349% | 0,119% | 0,000% |
| *Planctomycetes;c Planctomycea;o Gemmatales;f Isosphaeraceae;g Singulisphaera* | 0,806% | 0,097% | 0,000% |
| *Proteobacteria;c Alphaproteobacteria;o Caulobacterales;f Caulobacteraceae;g Asticcacaulis* | 0,004% | 0,009% | 0,000% |
| *Proteobacteria;c Alphaproteobacteria;o Rhizobiales;f ;g* | 0,067% | 0,011% | 0,000% |
| *Proteobacteria;c Alphaproteobacteria;o Rhizobiales;f Bradyrhizobiaceae;g* | 0,013% | 0,017% | 0,000% |
| *Proteobacteria;c Alphaproteobacteria;o Rhizobiales;f Brucellaceae;g Brucella* | 0,048% | 0,020% | 0,000% |
| *Proteobacteria;c Alphaproteobacteria;o Sphingomonadales;f ;g* | 0,010% | 0,012% | 0,000% |
| *Proteobacteria;c Alphaproteobacteria;o Sphingomonadales;f Sphingomonadaceae*;Other | 0,048% | 0,005% | 0,000% |
| *Proteobacteria;c Betaproteobacteria;o Burkholderiales;f Burkholderiaceae;g Cupriavidus* | 0,038% | 0,004% | 0,000% |
| *Proteobacteria;c Betaproteobacteria;o Burkholderiales;f Comamonadaceae;g Xenophilus* | 0,028% | 0,026% | 0,000% |
| *Proteobacteria;c Betaproteobacteria;o Burkholderiales;f Oxalobacteraceae;g* | 0,004% | 0,018% | 0,000% |
| *Proteobacteria;c Deltaproteobacteria;o Myxococcales;f Cystobacteraceae;g Stigmatella* | 0,009% | 0,007% | 0,000% |
| *Proteobacteria;c Gammaproteobacteria;o Chromatiales*;Other;Other | 0,023% | 0,003% | 0,000% |
| *Proteobacteria;c Gammaproteobacteria;o Oceanospirillales*;f 211ds20;g | 0,010% | 0,004% | 0,000% |
| *Proteobacteria;c Gammaproteobacteria;o Pseudomonadales;f Moraxellaceae;g Acinetobacter* | 0,113% | 0,041% | 0,000% |
| *Proteobacteria;c Gammaproteobacteria;o Xanthomonadales;f Xanthomonadaceae;g Dokdonella* | 0,058% | 0,014% | 0,000% |
| *Proteobacteria;c Gammaproteobacteria;o Xanthomonadales;f Xanthomonadaceae;g Frateuria* | 0,018% | 0,003% | 0,000% |
| *Verrucomicrobia;c Opitutae;o Opitutales;f Opitutaceae;g* | 0,009% | 0,004% | 0,000% |
| *Verrucomicrobia;c Opitutae;o Opitutales;f Opitutaceae;g Opitutus* | 0,013% | 0,002% | 0,000% |
| **Rare genera shared between the undiluted and the 10^4^-diluted microcosms** |  |  |  |
| *Acidobacteria*;c Sva0725;o Sva0725;f ;g | 0,004% | 0,000% | 0,004% |
| *Actinobacteria;c Actinobacteria;o Acidimicrobiales;*f CL500-29;g | 0,019% | 0,000% | 0,035% |
| CCM11b;c ;o ;f ;g | 0,009% | 0,000% | 0,004% |
| *Chlorobi*;c SJA-28;o ;f ;g | 0,013% | 0,000% | 0,039% |
| *Chloroflexi*;c Bljii12;o B07 WMSP1;f FFCH4570;g | 0,010% | 0,000% | 0,004% |
| *Proteobacteria;c Betaproteobacteria;o Burkholderiales;f Alcaligenaceae*;Other | 0,004% | 0,000% | 0,085% |
| *Proteobacteria;c Betaproteobacteria;o Nitrosomonadales;f Nitrosomonadaceae;g Nitrosospira* | 0,263% | 0,000% | 0,006% |
| *Proteobacteria;c Betaproteobacteria;o Rhodocyclales;f Rhodocyclaceae;g Methyloversatilis* | 0,010% | 0,000% | 0,003% |
| *Proteobacteria;c Deltaproteobacteria;o Myxococcales;f Cystobacteraceae;g* | 0,040% | 0,000% | 0,011% |
| *Proteobacteria;c Gammaproteobacteria;o Pseudomonadales;f Pseudomonadaceae;g Azotobacter* | 0,009% | 0,000% | 0,003% |
| **Rare genera shared between the 10^2^-diluted and the 10^4^-diluted microcosms** |  |  |  |
| *Actinobacteria;c Actinobacteria;o Actinomycetales;f Frankiaceae*;Other | 0,000% | 0,012% | 0,003% |
| *Actinobacteria;c Actinobacteria;o Actinomycetales;f Kineosporiaceae;g* | 0,000% | 0,005% | 0,018% |
| *Actinobacteria;c Actinobacteria;o Actinomycetales;f Micrococcaceae*;Other | 0,000% | 0,014% | 0,175% |
| *Actinobacteria;c Actinobacteria;o Actinomycetales;f Nocardiaceae;*Other | 0,000% | 0,025% | 0,018% |
| *Actinobacteria;c Actinobacteria;o Actinomycetales;f Promicromonosporaceae*;Other | 0,000% | 0,007% | 0,009% |
| *Actinobacteria;c Actinobacteria;o Actinomycetales;f Streptosporangiaceae;g Microbispora* | 0,000% | 0,003% | 0,015% |
| *Actinobacteria;c Actinobacteria;o Solirubrobacterales;f Patulibacteraceae;g* | 0,000% | 0,002% | 0,011% |
| *Cyanobacteria;c Chloroplast;o Chlorophyta;f Trebouxiophyceae;g* | 0,000% | 0,004% | 0,037% |
| *Firmicutes;c Bacilli;o Bacillales;f Bacillaceae;g Geobacillus* | 0,000% | 0,004% | 0,004% |
| *Firmicutes;c Bacilli;o Haloplasmatales;f Haloplasmataceae;g* | 0,000% | 0,038% | 0,027% |
| *Firmicutes;c Bacilli;o Turicibacterales;f Turicibacteraceae;g Turicibacter* | 0,000% | 0,004% | 0,003% |
| *Firmicutes;c Clostridia;o Clostridiales;f Catabacteriaceae;g* | 0,000% | 0,004% | 0,008% |
| *Firmicutes;c Clostridia;o Clostridiales;f Clostridiaceae;g* | 0,000% | 0,005% | 0,007% |
| *Firmicutes;c Clostridia;o Clostridiales;f ClostridialesFamilyXI,IncertaeSedis;g Sedimentibacter* | 0,000% | 0,003% | 0,019% |
| *Firmicutes;c Clostridia;o Clostridiales;f ClostridialesFamilyXI,IncertaeSedis;g Tissierella* | 0,000% | 0,006% | 0,035% |
| *Firmicutes;c Clostridia;o Clostridiales;f ClostridialesFamilyXIII,IncertaeSedis*;Other | 0,000% | 0,003% | 0,025% |
| *Firmicutes;c Clostridia;o Clostridiales;f Lachnospiraceae;g* | 0,000% | 0,009% | 0,007% |
| *Firmicutes;c Clostridia;o Clostridiales;f Lachnospiraceae;g Clostridium* | 0,000% | 0,008% | 0,008% |
| *Firmicutes;c Clostridia;o Clostridiales;f Lachnospiraceae;g Epulopiscium* | 0,000% | 0,008% | 0,032% |
| *Firmicutes;c Clostridia;o Clostridiales;f Peptococcaceae*;Other | 0,000% | 0,022% | 0,083% |
| *Firmicutes;c Clostridia;o Clostridiales;f Peptococcaceae;g* | 0,000% | 0,009% | 0,007% |
| *Firmicutes;c Clostridia;o Clostridiales;f Ruminococcaceae*;Other | 0,000% | 0,006% | 0,033% |
| *Firmicutes;c Clostridia;o Clostridiales;f Veillonellaceae;g Thermosinus* | 0,000% | 0,004% | 0,004% |
| *Proteobacteria;c Alphaproteobacteria;o Rhizobiales;f Methylocystaceae;g Methylosinus* | 0,000% | 0,005% | 0,017% |
| *Proteobacteria;c Alphaproteobacteria;o Sphingomonadales;f Sphingomonadaceae;g* | 0,000% | 0,004% | 0,008% |
| *Proteobacteria;c Deltaproteobacteria;o Myxococcales;f Haliangiaceae*;Other | 0,000% | 0,004% | 0,021% |
| *Proteobacteria;c Gammaproteobacteria;o Legionellales;f Coxiellaceae;g Aquicella* | 0,000% | 0,023% | 0,039% |
| SC3;c ;o ;f ;g | 0,000% | 0,002% | 0,004% |
| TM6;c SBRH58;o ;f ;g | 0,000% | 0,007% | 0,011% |
| TM6;c SJA-4;o ;f ;g | 0,000% | 0,010% | 0,014% |
| WS3;c PRR-12;o LD1-PA13;f ;g | 0,000% | 0,005% | 0,011% |
| **Rare genera detected in all microcosms** |  |  |  |
| *Acidobacteria;c ;o ;f ;g* | 0,067% | 0,005% | 0,023% |
| *Acidobacteria;c ;o ;f Koribacteraceae*;Other | 0,014% | 0,018% | 0,018% |
| *Acidobacteria;c ;o ;f Koribacteraceae;g CandidatusKoribacter* | 0,081% | 0,010% | 0,014% |
| *Acidobacteria;c Acidobacteria;o Acidobacteriales;f ;g* | 0,606% | 0,110% | 0,033% |
| *Acidobacteria;c Acidobacteria;o Acidobacteriales;f Acidobacteriaceae;g Edaphobacter* | 0,167% | 0,217% | 0,004% |
| *Acidobacteria*;c iii1-8;o 32-20;f ;g | 0,206% | 0,165% | 0,391% |
| *Actinobacteria;c Actinobacteria*;o ;f ;g | 0,018% | 0,017% | 0,037% |
| *Actinobacteria;c Actinobacteria;o Acidimicrobiales*;Other;Other | 0,152% | 0,055% | 0,293% |
| *Actinobacteria;c Actinobacteria;o Acidimicrobiales;f ;g* | 0,019% | 0,010% | 0,038% |
| *Actinobacteria;c Actinobacteria;o Acidimicrobiales*;f AKIW874;g | 0,004% | 0,017% | 0,050% |
| *Actinobacteria;c Actinobacteria;o Acidimicrobiales*;f EB1017;g | 0,121% | 0,355% | 0,581% |
| *Actinobacteria;c Actinobacteria;o Acidimicrobiales;f Iamiaceae;g* | 0,036% | 0,043% | 0,131% |
| *Actinobacteria;c Actinobacteria;o Acidimicrobiales;f Iamiaceae;g Iamia* | 0,047% | 0,004% | 0,051% |
| *Actinobacteria;c Actinobacteria;o Actinomycetales;f Actinosynnemataceae;g* | 0,027% | 0,278% | 0,059% |
| *Actinobacteria;c Actinobacteria;o Actinomycetales;f Intrasporangiaceae*;Other | 0,064% | 0,010% | 0,024% |
| *Actinobacteria;c Actinobacteria;o Actinomycetales;f Intrasporangiaceae;g Phycicoccus* | 0,023% | 0,035% | 0,074% |
| *Actinobacteria;c Actinobacteria;o Actinomycetales;f Intrasporangiaceae;g Terracoccus* | 0,621% | 0,422% | 0,474% |
| *Actinobacteria;c Actinobacteria;o Actinomycetales;f Microbacteriaceae;g Frigoribacterium* | 0,009% | 0,005% | 0,003% |
| *Actinobacteria;c Actinobacteria;o Actinomycetales;f Microbacteriaceae;g Microbacterium* | 0,027% | 0,007% | 0,004% |
| *Actinobacteria;c Actinobacteria;o Actinomycetales;f Micrococcaceae;g Arthrobacter* | 0,013% | 0,012% | 0,068% |
| *Actinobacteria;c Actinobacteria;o Actinomycetales;f Micromonosporaceae*;Other | 0,235% | 0,285% | 0,152% |
| *Actinobacteria;c Actinobacteria;o Actinomycetales;f Micromonosporaceae;g Micromonospora* | 0,296% | 0,345% | 0,143% |
| *Actinobacteria;c Actinobacteria;o Actinomycetales;f Mycobacteriaceae;g Mycobacterium* | 0,036% | 0,029% | 0,082% |
| *Actinobacteria;c Actinobacteria;o Actinomycetales;f Nocardiaceae;g Rhodococcus* | 0,396% | 0,292% | 0,312% |
| *Actinobacteria;c Actinobacteria;o Actinomycetales;f Nocardioidaceae*;Other | 0,556% | 0,411% | 0,403% |
| *Actinobacteria;c Actinobacteria;o Actinomycetales;f Nocardioidaceae;g* | 0,019% | 0,065% | 0,079% |
| *Actinobacteria;c Actinobacteria;o Actinomycetales;f Nocardioidaceae;g Actinopolymorpha* | 0,056% | 0,147% | 0,022% |
| *Actinobacteria;c Actinobacteria;o Actinomycetales;f Nocardioidaceae;g Marmoricola* | 0,229% | 0,261% | 0,246% |
| *Actinobacteria;c Actinobacteria;o Actinomycetales;f Nocardioidaceae;g Pimelobacter* | 0,013% | 0,006% | 0,003% |
| *Actinobacteria;c Actinobacteria;o Actinomycetales;f Promicromonosporaceae;g Promicromonospora* | 0,434% | 0,405% | 0,112% |
| *Actinobacteria;c Actinobacteria;o Actinomycetales;f Propionibacteriaceae;g* | 0,010% | 0,011% | 0,007% |
| *Actinobacteria;c Actinobacteria;o Actinomycetales;f Pseudonocardiaceae*;Other | 0,014% | 0,003% | 0,006% |
| *Actinobacteria;c Actinobacteria;o Actinomycetales;f Pseudonocardiaceae;g Amycolatopsis* | 0,019% | 0,131% | 0,083% |
| *Actinobacteria;c Actinobacteria;o Actinomycetales;f Pseudonocardiaceae;g Pseudonocardia* | 0,125% | 0,113% | 0,179% |
| *Actinobacteria;c Actinobacteria;o Actinomycetales;f Pseudonocardiaceae;g Saccharomonospora* | 0,018% | 0,003% | 0,011% |
| *Actinobacteria;c Actinobacteria;o Actinomycetales;f Pseudonocardiaceae;g Saccharopolyspora* | 0,170% | 0,581% | 0,039% |
| *Actinobacteria;c Actinobacteria;o Actinomycetales;f Streptomycetaceae;g Kitasatospora* | 0,036% | 0,142% | 0,072% |
| *Actinobacteria;c Actinobacteria;o Actinomycetales;f Streptosporangiaceae;g Nonomuraea* | 0,027% | 0,107% | 0,033% |
| *Actinobacteria;c Actinobacteria;o Actinomycetales;f Streptosporangiaceae;g Streptosporangium* | 0,009% | 0,047% | 0,007% |
| *Actinobacteria;c Actinobacteria;o Actinomycetales;f Thermomonosporaceae;g Actinomadura* | 0,069% | 0,136% | 0,014% |
| *Actinobacteria;c Actinobacteria;o Solirubrobacterales;f* *Solirubrobacteraceae;g* | 0,019% | 0,019% | 0,014% |
| *Armatimonadetes*;c SJA-176;o ;f ;g | 0,004% | 0,004% | 0,015% |
| *Bacteroidetes;c Flavobacteria;o Flavobacteriales;f Flavobacteriaceae;g Flavobacterium* | 0,044% | 0,019% | 0,006% |
| *Bacteroidetes;c Sphingobacteria;o Sphingobacteriales*;f ;Other | 0,343% | 0,115% | 0,030% |
| *Bacteroidetes;c Sphingobacteria;o Sphingobacteriales;f ;g Chitinophaga* | 0,343% | 0,288% | 0,460% |
| *Bacteroidetes;c Sphingobacteria;o Sphingobacteriales;f ;g Flavisolibacter* | 0,073% | 0,005% | 0,012% |
| *Bacteroidetes;c Sphingobacteria;o Sphingobacteriales;f Flexibacteraceae;g Cytophaga* | 0,324% | 0,200% | 0,087% |
| *Bacteroidetes;c Sphingobacteria;o Sphingobacteriales;f Sphingobacteriaceae;g Pedobacter* | 0,715% | 0,204% | 0,008% |
| *Chloroflexi;c Ktedonobacteria*;o ;f ;g | 0,056% | 0,037% | 0,031% |
| *Chloroflexi*;c SOGA31;o ;f ;g | 0,049% | 0,084% | 0,079% |
| *Chloroflexi*;c TK17;o ;f ;g | 0,019% | 0,031% | 0,013% |
| *Chloroflexi;c Thermomicrobia;*o HN1-15;f ;g | 0,185% | 0,061% | 0,024% |
| *Crenarchaeota*;c C2;o pGrfC26;f ;g | 0,009% | 0,020% | 0,034% |
| *Crenarchaeota;c Thaumarchaeota;o Nitrososphaerales;f Nitrososphaeraceae;g CandidatusNitrososphaera* | 0,046% | 0,066% | 0,102% |
| *Firmicutes*;Other;Other;Other;Other | 0,049% | 0,074% | 0,213% |
| *Firmicutes;c Bacilli*;Other;Other;Other | 0,053% | 0,031% | 0,112% |
| *Firmicutes;c Bacilli;o Bacillales;f Alicyclobacillaceae;g Alicyclobacillus* | 0,004% | 0,018% | 0,046% |
| *Firmicutes;c Bacilli;o Bacillales;f Bacillaceae*;Other | 0,023% | 0,065% | 0,093% |
| *Firmicutes;c Bacilli;o Bacillales;f Paenibacillaceae;g Ammoniphilus* | 0,128% | 0,293% | 0,517% |
| *Firmicutes;c Bacilli;o Bacillales;f Paenibacillaceae;g Brevibacillus* | 0,004% | 0,008% | 0,015% |
| *Firmicutes;c Bacilli;o Bacillales;f Paenibacillaceae;g Cohnella* | 0,136% | 0,088% | 0,145% |
| *Firmicutes;c Bacilli;o Bacillales;f Paenibacillaceae;g Paenibacillus* | 0,520% | 0,529% | 0,453% |
| *Firmicutes;c Bacilli;o Bacillales;f Planococcaceae;g* | 0,093% | 0,340% | 0,454% |
| *Firmicutes;c Bacilli;o Bacillales;f Planococcaceae;g Kurthia* | 0,018% | 0,016% | 0,021% |
| *Firmicutes;c Bacilli;o Bacillales;f Planococcaceae;g Paenisporosarcina* | 0,014% | 0,012% | 0,007% |
| *Firmicutes;c Bacilli;o Bacillales;f Planococcaceae;g Rummeliibacillus* | 0,018% | 0,010% | 0,008% |
| *Firmicutes;c Bacilli;o Bacillales;f Planococcaceae;g Solibacillus* | 0,166% | 0,133% | 0,397% |
| *Firmicutes;c Bacilli;o Bacillales;f Planococcaceae;g Sporosarcina* | 0,428% | 0,494% | 0,219% |
| *Firmicutes;c Bacilli;o Bacillales;f Planococcaceae;g Ureibacillus* | 0,009% | 0,002% | 0,026% |
| *Firmicutes;c Bacilli;o Bacillales;f Thermoactinomycetaceae*;Other | 0,027% | 0,070% | 0,100% |
| *Firmicutes;c Bacilli;o Bacillales;f Thermoactinomycetaceae;g Shimazuella* | 0,179% | 0,344% | 0,066% |
| *Firmicutes;c Bacilli;o Turicibacterales;f Turicibacteraceae*;Other | 0,069% | 0,110% | 0,261% |
| *Firmicutes;c Bacilli;o Turicibacterales;f Turicibacteraceae;g* | 0,040% | 0,080% | 0,173% |
| *Firmicutes;c Clostridia*;Other;Other;Other | 0,009% | 0,031% | 0,110% |
| *Firmicutes;c Clostridia;o Clostridiales*;f BSV43;g | 0,018% | 0,003% | 0,016% |
| *Firmicutes;c Clostridia;o Clostridiales;f Clostridiaceae*;Other | 0,082% | 0,193% | 0,427% |
| *Firmicutes;c Clostridia;o Clostridiales;f Clostridiaceae;g Alkaliphilus* | 0,022% | 0,029% | 0,017% |
| *Firmicutes;c Clostridia;o Clostridiales;f Clostridiaceae;g Caloramator* | 0,018% | 0,044% | 0,088% |
| *Firmicutes;c Clostridia;o Clostridiales;f Lachnospiraceae;*Other | 0,111% | 0,179% | 0,349% |
| *Firmicutes;c Clostridia;o Clostridiales;f Lachnospiraceae;g Anaerostipes* | 0,027% | 0,137% | 0,176% |
| *Firmicutes;c Clostridia;o Clostridiales;f Peptococcaceae;g Desulfitobacterium* | 0,065% | 0,142% | 0,221% |
| *Firmicutes;c Clostridia;o Clostridiales;f Peptococcaceae;g Desulfosporosinus* | 0,128% | 0,437% | 0,846% |
| *Firmicutes;c Clostridia;o Clostridiales;f Peptostreptococcaceae*;Other | 0,031% | 0,027% | 0,115% |
| *Firmicutes;c Clostridia;o Clostridiales;f Peptostreptococcaceae;g Tepidibacter* | 0,027% | 0,134% | 0,149% |
| *Firmicutes;c Clostridia;o Clostridiales;f Ruminococcaceae;g* | 0,019% | 0,011% | 0,025% |
| *Firmicutes;c Clostridia;o Clostridiales;f Ruminococcaceae;g Acetivibrio* | 0,028% | 0,130% | 0,221% |
| *Firmicutes;c Clostridia;o Clostridiales;f Ruminococcaceae;g Ruminococcus* | 0,013% | 0,009% | 0,011% |
| *Firmicutes;c Clostridia;o Clostridiales;f Veillonellaceae*;Other | 0,131% | 0,260% | 0,314% |
| *Firmicutes;c Clostridia;o Clostridiales;f Veillonellaceae;g* | 0,010% | 0,040% | 0,100% |
| *Firmicutes;c Clostridia;o Clostridiales;f Veillonellaceae;g Desulfosporomusa* | 0,018% | 0,057% | 0,047% |
| *Firmicutes;c Clostridia;o Clostridiales;f Veillonellaceae;g Sporotalea* | 0,070% | 0,158% | 0,256% |
| *Firmicutes;c Clostridia;o Halanaerobiales;f Halanaerobiaceae;g* | 0,023% | 0,070% | 0,111% |
| *Firmicutes;c Clostridia*;o OPB54;f ;g | 0,032% | 0,042% | 0,102% |
| *Gemmatimonadetes;c Gemmatimonadetes;o Gemmatimonadales;f ;g* | 0,414% | 0,116% | 0,231% |
| *Nitrospirae;c Nitrospira;o Nitrospirales;f Nitrospiraceae;g Nitrospira* | 0,074% | 0,026% | 0,094% |
| *Nitrospirae;c Nitrospira;o Nitrospirales;f Thermodesulfovibrionaceae*;g GOUTA19 | 0,019% | 0,024% | 0,023% |
| *Planctomycetes;c Phycisphaerae;o ;f ;g* | 0,123% | 0,005% | 0,008% |
| *Planctomycetes;c Planctomycea;o Planctomycetales;f Planctomycetaceae;g Planctomyces* | 0,130% | 0,021% | 0,003% |
| *Proteobacteria*;Other;Other;Other;Other | 0,350% | 0,173% | 0,137% |
| *Proteobacteria;c Alphaproteobacteria*;Other;Other;Other | 0,188% | 0,182% | 0,110% |
| *Proteobacteria;c Alphaproteobacteria;o ;f ;g* | 0,239% | 0,107% | 0,032% |
| *Proteobacteria;c Alphaproteobacteria;o Caulobacterales;f Caulobacteraceae*;Other | 0,081% | 0,074% | 0,003% |
| *Proteobacteria;c Alphaproteobacteria;o Caulobacterales;f Caulobacteraceae;g Caulobacter* | 0,101% | 0,144% | 0,078% |
| *Proteobacteria;c Alphaproteobacteria;o Caulobacterales;f Caulobacteraceae;g Phenylobacterium* | 0,119% | 0,144% | 0,099% |
| *Proteobacteria;c Alphaproteobacteria;o Rhizobiales*;f ;Other | 0,229% | 0,053% | 0,036% |
| *Proteobacteria;c Alphaproteobacteria;o Rhizobiales;f ;g Nordella* | 0,352% | 0,061% | 0,021% |
| *Proteobacteria;c Alphaproteobacteria;o Rhizobiales;f Beijerinckiaceae*;Other | 0,054% | 0,025% | 0,008% |
| *Proteobacteria;c Alphaproteobacteria;o Rhizobiales;f Beijerinckiaceae;g* | 0,199% | 0,059% | 0,053% |
| *Proteobacteria;c Alphaproteobacteria;o Rhizobiales;f Bradyrhizobiaceae;g Afipia* | 0,370% | 0,682% | 0,313% |
| *Proteobacteria;c Alphaproteobacteria;o Rhizobiales;f Bradyrhizobiaceae;g Balneimonas* | 0,151% | 0,078% | 0,026% |
| *Proteobacteria;c Alphaproteobacteria;o Rhizobiales;f Bradyrhizobiaceae;g Rhodopseudomonas* | 0,180% | 0,116% | 0,008% |
| *Proteobacteria;c Alphaproteobacteria;o Rhizobiales;f Brucellaceae;g Ochrobactrum* | 0,360% | 0,327% | 0,043% |
| *Proteobacteria;c Alphaproteobacteria;o Rhizobiales;f Hyphomicrobiaceae*;Other | 0,147% | 0,090% | 0,138% |
| *Proteobacteria;c Alphaproteobacteria;o Rhizobiales;f Hyphomicrobiaceae;g* | 0,004% | 0,003% | 0,006% |
| *Proteobacteria;c Alphaproteobacteria;o Rhizobiales;f Hyphomicrobiaceae;g Hyphomicrobium* | 0,155% | 0,210% | 0,364% |
| *Proteobacteria;c Alphaproteobacteria;o Rhizobiales;f Hyphomicrobiaceae;g Pedomicrobium* | 0,398% | 0,573% | 1,105% |
| *Proteobacteria;c Alphaproteobacteria;o Rhizobiales;f Hyphomicrobiaceae;g Rhodomicrobium* | 0,027% | 0,029% | 0,094% |
| *Proteobacteria;c Alphaproteobacteria;o Rhizobiales;f Phyllobacteriaceae*;Other | 0,415% | 0,287% | 0,154% |
| *Proteobacteria;c Alphaproteobacteria;o Rhizobiales;f Phyllobacteriaceae;g* | 0,041% | 0,027% | 0,204% |
| *Proteobacteria;c Alphaproteobacteria;o Rhizobiales;f Phyllobacteriaceae;g Aminobacter* | 0,029% | 0,023% | 0,022% |
| *Proteobacteria;c Alphaproteobacteria;o Rhizobiales;f Rhizobiaceae;g Agrobacterium* | 0,526% | 0,263% | 0,356% |
| *Proteobacteria;c Alphaproteobacteria;o Rhizobiales;f Rhizobiaceae;g Kaistia* | 0,168% | 0,028% | 0,008% |
| *Proteobacteria;c Alphaproteobacteria;o Rhizobiales;f Xanthobacteraceae;g* | 0,041% | 0,022% | 0,004% |
| *Proteobacteria;c Alphaproteobacteria;o Rhizobiales;f Xanthobacteraceae;g Labrys* | 0,368% | 0,207% | 0,090% |
| *Proteobacteria;c Alphaproteobacteria;o Rhodospirillales;f Acetobacteraceae*;Other | 0,068% | 0,091% | 0,003% |
| *Proteobacteria;c Alphaproteobacteria;o Rhodospirillales;f Acetobacteraceae;g* | 0,035% | 0,002% | 0,021% |
| *Proteobacteria;c Alphaproteobacteria;o Rhodospirillales;f Rhodospirillaceae*;Other | 0,023% | 0,022% | 0,007% |
| *Proteobacteria;c Alphaproteobacteria;o Rhodospirillales;f Rhodospirillaceae;g Inquilinus* | 0,032% | 0,034% | 0,017% |
| *Proteobacteria;c Alphaproteobacteria;o Sphingomonadales*;Other;Other | 0,056% | 0,030% | 0,007% |
| *Proteobacteria;c Alphaproteobacteria;o Sphingomonadales;f Sphingomonadaceae;g Kaistobacter* | 0,013% | 0,016% | 0,006% |
| *Proteobacteria;c Alphaproteobacteria;o Sphingomonadales;f Sphingomonadaceae;g Sphingomonas* | 0,055% | 0,009% | 0,007% |
| *Proteobacteria;c Alphaproteobacteria;o Sphingomonadales;f Sphingomonadaceae;g Sphingopyxis* | 0,036% | 0,028% | 0,014% |
| *Proteobacteria;c Betaproteobacteria*;Other;Other;Other | 0,118% | 0,021% | 0,154% |
| *Proteobacteria;c Betaproteobacteria;o ;f ;g* | 0,269% | 0,046% | 0,094% |
| *Proteobacteria;c Betaproteobacteria;o Burkholderiales*;Other;Other | 0,625% | 0,266% | 0,137% |
| *Proteobacteria;c Betaproteobacteria;o Burkholderiales*;f ;Other | 0,102% | 0,055% | 0,003% |
| *Proteobacteria;c Betaproteobacteria;o Burkholderiales;f ;g Methylibium* | 0,055% | 0,025% | 0,007% |
| *Proteobacteria;c Betaproteobacteria;o Burkholderiales;f Alcaligenaceae;g* | 0,181% | 0,012% | 0,046% |
| *Proteobacteria;c Betaproteobacteria;o Burkholderiales;f Alcaligenaceae;g Achromobacter* | 0,054% | 0,070% | 0,007% |
| *Proteobacteria;c Betaproteobacteria;o Burkholderiales;f Burkholderiaceae;g Burkholderia* | 0,583% | 0,078% | 0,080% |
| *Proteobacteria;c Betaproteobacteria;o Burkholderiales;f Comamonadaceae;g Polaromonas* | 0,292% | 0,252% | 0,007% |
| *Proteobacteria;c Betaproteobacteria;o Burkholderiales;f Oxalobacteraceae*;Other | 0,056% | 0,019% | 0,008% |
| *Proteobacteria;c Betaproteobacteria;o Rhodocyclales;f ;g* | 0,294% | 0,026% | 0,045% |
| *Proteobacteria;c Deltaproteobacteria*;Other;Other;Other | 0,052% | 0,021% | 0,040% |
| *Proteobacteria;c Deltaproteobacteria;o ;f ;g* | 0,004% | 0,038% | 0,064% |
| *Proteobacteria;c Deltaproteobacteria;o Bdellovibrionales;f Bdellovibrionaceae;g* | 0,032% | 0,029% | 0,085% |
| *Proteobacteria;c Deltaproteobacteria;o Desulfuromonadales;f Geobacteraceae;g Geobacter* | 0,026% | 0,002% | 0,015% |
| *Proteobacteria;c Deltaproteobacteria*;o MIZ46;f ;g | 0,013% | 0,047% | 0,081% |
| *Proteobacteria;c Deltaproteobacteria;o Myxococcales*;Other;Other | 0,500% | 0,293% | 0,365% |
| *Proteobacteria;c Deltaproteobacteria;o Myxococcales;f ;g* | 0,244% | 0,166% | 0,124% |
| *Proteobacteria;c Deltaproteobacteria;o Myxococcales;f Cystobacteraceae;*Other | 0,105% | 0,054% | 0,167% |
| *Proteobacteria;c Deltaproteobacteria;o Myxococcales;f Haliangiaceae;g* | 0,366% | 0,471% | 0,761% |
| *Proteobacteria;c Deltaproteobacteria;o Myxococcales;f Myxococcaceae;g Anaeromyxobacter* | 0,037% | 0,022% | 0,128% |
| *Proteobacteria;c Deltaproteobacteria;o Myxococcales;f Nannocystaceae;g Plesiocystis* | 0,004% | 0,002% | 0,003% |
| *Proteobacteria;c Deltaproteobacteria;o Myxococcales;f Polyangiaceae;g* | 0,070% | 0,106% | 0,254% |
| *Proteobacteria;c Deltaproteobacteria*;o NB1-j;Other;Other | 0,010% | 0,003% | 0,007% |
| *Proteobacteria;c Gammaproteobacteria*;Other;Other;Other | 0,051% | 0,032% | 0,018% |
| *Proteobacteria;c Gammaproteobacteria;o Chromatiales;f ;g* | 0,037% | 0,008% | 0,045% |
| *Proteobacteria;c Gammaproteobacteria;o Chromatiales;f Sinobacteraceae;*Other | 0,273% | 0,078% | 0,066% |
| *Proteobacteria;c Gammaproteobacteria;o Pseudomonadales;f Pseudomonadaceae*;Other | 0,027% | 0,002% | 0,012% |
| *Proteobacteria;c Gammaproteobacteria;o Xanthomonadales;f Xanthomonadaceae*;Other | 0,381% | 0,297% | 0,047% |
| *Proteobacteria;c Gammaproteobacteria;o Xanthomonadales;f Xanthomonadaceae;g* | 0,093% | 0,062% | 0,022% |
| *Proteobacteria;c Gammaproteobacteria;o Xanthomonadales;f Xanthomonadaceae;g Lysobacter* | 0,917% | 0,318% | 0,011% |
| *Proteobacteria;c Gammaproteobacteria;o Xanthomonadales;f Xanthomonadaceae;g Pseudoxanthomonas* | 0,235% | 0,068% | 0,063% |
| *Proteobacteria;c Gammaproteobacteria;o Xanthomonadales;f Xanthomonadaceae;g Rhodanobacter* | 0,048% | 0,002% | 0,101% |
| *Proteobacteria;c Gammaproteobacteria;o Xanthomonadales;f Xanthomonadaceae;g Thermomonas* | 0,516% | 0,882% | 0,119% |
| SPAM;c 0319-6G9;o ;f ;g | 0,009% | 0,006% | 0,004% |
| *Tenericutes*;c ML615J-28;o ;f ;g | 0,024% | 0,009% | 0,044% |
| *Verrucomicrobia;c Spartobacteria;o Spartobacteriales;f Spartobacteriaceae;g CandidatusXiphinematobacter* | 0,023% | 0,004% | 0,008% |
| *Verrucomicrobia;c Spartobacteria;o Spartobacteriales;f Spartobacteriaceae;g Chthoniobacter* | 0,182% | 0,029% | 0,007% |
| *Verrucomicrobia;c Spartobacteria;o Spartobacteriales;f Spartobacteriaceae*;g MC18 | 0,019% | 0,004% | 0,004% |
| WS3;c PRR-12;o Sediment-1;Other;Other | 0,048% | 0,042% | 0,123% |
